# Emotional Interaction in Road Traffic Injury: A Qualitative Study On People With Spinal Cord Injury

**DOI:** 10.25122/jml-2019-0078

**Published:** 2019

**Authors:** Elham Sepahvand, Hamidreza Khankeh, Mohammadali Hosseini, Behnam Akhbari

**Affiliations:** 1. Department of Nursing, University of Social Welfare and Rehabilitation Sciences, Tehran, Iran; 2. Research center of health in emergency and disasters, University of social welfare and rehabilitation sciences, Tehran, Iran; 3. Department of Clinical Science and Education, Karolinska Institutet, Södersjukhuset, Stockholm, Sweden; 4. Department of Physiotherapy, University of Social Welfare and Rehabilitation Sciences, Tehran, Iran

**Keywords:** Emotional interaction, Qualitative study, Content analysis, Spinal cord injury, Road traffic injury

## Abstract

The injury management in the acute phase of spinal cord injury starts at the accident scene and focuses on preventing and reducing secondary damages. The road traffic injured patients are mostly transferred by relatives, untrained laypeople, and the drivers of heavy vehicles. The current study explored the experience of people with spinal cord injury in the accident scene.

This was a qualitative content analysis study using the semi-structured interviewing method with an interview guide for data collection. Purposive sampling method was performed within ten months until data saturation. We used the constant comparative approach recommended by Corbin and Strauss (2015). In total, 15 people with spinal cord injury and bystanders participated in this study.

The central theme extracted in this study was “emotional interaction” that referred to the emotional reactions in managing road traffic victims. Two main categories of “emotional intervention” with “emotional atmosphere,” “desperation,” “rescue efforts,” subcategories and “scene shock” with “unplanned intervention,” “emotional behavior,” “emotional decisions,” and “emotional involvement,” subcategories were classified.

The emotional atmosphere of the scene and stress level of the victim and the relatives, and the insistence of the victim to escape from the harsh condition have caused those lacking medical knowledge and expertise to transfer the patient unsafely. This resulted in secondary damages, like aggravated spinal cord injury or even caused the spinal cord injury.

## Introduction

Traumatic injuries caused by traffic collisions are still a significant public health problem, especially in low-income countries. According to global statistics, about 27.1 million deaths and nearly 50 million injuries occur annually following road traffic accidents [1]. The World Health Organization (WHO) recognizes car accidents as the ninth cause of disability. According to the WHO, by 2020, car accidents will be recognized as the third cause of disability after heart disease and depression [2]. In Iran, as the first health problem, accidents waste more than one million and 200000 years of life annually [3]. Traffic collisions cause multiple neuromusculoskeletal disorders among injured people. Of all problems, central nervous system injuries, including damages to the brain and spinal cord, are influential. This is due to the severity of such injuries and their long-term effects [4]. Spinal Cord Injuries (SCIs) are devastating and rank among the major public health problems. They can lead to the dysfunction of motor and autonomic systems, as well as permanent disabilities [5]. Also, SCIs contribute to pain and disability for the upcoming years following the incident [4]. Most of the problems mentioned above affect the young, and this adds up individuals’ mental stress and economic pressure on the community [6] Over 60% of spinal cord injuries afflict young men aged 15 to 35 who are at an age that allows earning money and whose disability would bring about economic hazards to the society, leading to a significant financial burden. The caring costs of spinal cord injuries are also significant [7]. The injured management in the acute phase of spinal cord injuries has been started at the accident scene and focuses on preventing and reducing secondary damages[1, 7]. Therefore, pre-hospital management at the accident scene should include rapid assessment of the injured, restoration of vital functions (airway, breathing, and circulation), more precise secondary examination, and eventually initiate a transfer to the trauma center [8].

Haghparast et al. argued that the management of the accident scene could have a significant impact on mortality and disability after the incident [9]. Most deaths occur on the accident scene and during the transfer of the injured person from the accident scene, which indicates the importance of care in the scene and the transferring process [10].

Nevertheless, few studies on the causes and how to provide services by pre-hospital nurses suggest that pre-hospital care is inappropriate and inadequate in developing countries. In these countries, only a small number of injured people have received primary care at the accident scene and were not even transferred to the hospital by ambulance. The injured patients are mostly transferred by relatives, untrained laypeople, and drivers of heavy vehicles [9, 11]. Several studies have also been conducted in Iran in this field, suggesting that various causes interfere with the injury in traffic accidents. These studies in the field of pre-hospital management revealed that setbacks such as involvement of laypeople in the accident scene, increased workload of staff and actions related to the injured patients and management issues, including the lack of adequate communication technology, prevented the delivery of appropriate pre-hospital services to the victims in the post-accident phase, which could affect their mortality and disability [12-14]. According to a study conducted by Khankeh et al., the essential elements in the pre-hospital care system are human factors, communication, and interactions that occur at the scene of the incident [1]. Consequently, it may be claimed that pre-hospital care at the accident scene involves a series of human interactions that occur in a specific context and may lead to injury in the victims [15]. The interaction of people in an accident scene in emergency situations is one of the important phenomena that may occur at the scene [16].

At the scene of the incident, people behave according to the perception of the environment that may be risky and could result in injury or cause no danger. Understanding the existing situation is necessary in order to take steps to strengthen the pre-hospital care system and ultimately reduce the burden of injuries and other conditions requiring emergency services. Hence, we were seeking to answer the following questions:

• What is the management of spinal cord injuries at the site of the accident?
• What happens at the scene of the incident that results in secondary damages?

## Material and Methods

The design of this study was a qualitative study that was performed by using the qualitative content analysis method. Content analysis is a valid research method for data analysis [17]. The participants included 16 people, 9 of whom have suffered spinal cord injuries, and 7 bystanders of traffic incidents. The participants were referrals of the Rofeideh Rehabilitation Hospital and Shahid Jalaypour’s Spinal Cord Injury Center in Tehran, Iran. The presence of the researcher as a clinical nurse and instructor, familiarity with the environment, and having access to knowledgeable individuals have been the reasons for the selection of these centers.

The purposeful sampling was used in the present study. In order to consider the maximum variation in the samples, attempts have been made to sample subjects with spinal cord injuries experiencing different conditions. Sampling was performed within ten months until data saturation was accomplished.

A semi-structured interview was conducted using template questions for collecting data. In the first step, the researcher described the purpose of the research to the participants; consequently, if they wished to participate, they were interviewed after obtaining the participants’ freely given informed consent in writing. The interviews were conducted using a tape recorder. Saturation data in the present study was carried out from the 14th interview. The question of the contributors was as follows: “Please describe the scene of the accident from the time of the accident to the time of arrival to the hospital.” In each interview, there were also follow-up questions such as “Can you explain more about this?”, and “What do you mean by what you said?” In the end, the participants were invited to express any other facts regarding the accident. The next questions were designed based on the participants’ responses and the interview process. Additionally, the researcher used other methods of data collection, including a focus group interview. The duration of the interview was 40 minutes on average.

Since the current study was part of a greater study of the grounded theory approach by Strauss and Corbin, data analysis was performed utilizing constant comparative analysis [17]. Each interview was read and reviewed several times, and after gaining a general sense, the analysis of the data was conducted using an inductive approach (that is, without the researcher’s preconceived image). Important clauses were defined by line-by-line reading, and important parts were underlined to be distinguished from other parts. Afterward, the initial codes or the concept of the first, second, and third level were created. In the end, the content contained in the data was introduced as the “theme” by comparing the category and subcategory with each other and deep and accurate reflection.

Lincoln and Guba’s evaluative criteria [18] were applied to determine the accuracy and reliability of the data. Trustworthiness of the researcher was confirmed through (a long-term contact with participants and attracting their trust) triangulation, exploiting the interview guide, allocating sufficient time for conducting interviews, constant comparison analysis of data and categories in terms of similarities and differences, re-reviewing the findings with the participants, providing detailed analysis of the data, as well as deep and rich descriptions of the research for the readers.

The Ethics Committee of the University of Social Welfare and Rehabilitation Sciences gave permission to conduct this research (IR.USWR.REC.1395.399).

## Results

A total of 15 patients with spinal cord injuries and companions or bystanders were interviewed. The characteristics of the participants are shown in Table 1. The main source extracted in this study was “emotional interaction” that referred to the emotional reactions in dealing with the injured patient in a traffic incident and was classified into two main categories: emotional intervention and scene shock. The two mentioned factors totally explained the emotional interaction (Table 2).

**Table 1:** Characteristics of participants.

Rank	Participant	Age	Sex	Duration of interview	Interview place
1	C1	32	Male	35	Hospital
2	C2	18	Male	40	Hospital
3	C3	50	Male	25	Hospital
4	C4	22	Male	30	Hospital
5	C5	22	Female	30	Rehabilitation center
6	C6	36	Female	28	Rehabilitation center
7	C7	19	Female	35	Rehabilitation center
8	H1	28	Male	36	Hospital
9	H2	50	Male	40	Rehabilitation center
10	H3	28	Female	28	Hospital
11	H4	26	Female	29	Rehabilitation center
12	C8	32	Female	31	Rehabilitation center
13	C9	18	Male	33	Hospital
14	L1	50	Male	36	Rehabilitation center
15	L2	33	Male	27	Rehabilitation center

**Table 2:** Codes, subcategory, category, and extracted themes.

**Themes (the fourth level concept)**	**Category (the third level concept)**	**Subcategory (the second level concept)**	**Primary codes (the first level concept)**
Emotional interaction	Emotional intervention	Unplanned interventions	The lack of protection
			Unprotected transfer
			Unstable condition of the injured
			Taking the injured away from the incident scene
		Emotional behaviors	Violence to the emergency forces
			Insisting on pulling the injured
			Insisting on a quick rescue
			Emotional dissonance
			Insisting on transferring the injured
		Emotional decisions	Hastiness
			The lack of emergency contact
			Transferring the injured with private cars
			Taking non-secure transfer actions
		Emotional involvement	Intervention for rescuers
			Rescue contribution without permission
			Gathering in the scene and blocking the way
			Intervention with a sense of being a savior
	Scene Shock	Rescue efforts	Fear of secondary incidents
			The urge to rescue
			Unstable position
			Blaming oneself
		Desperation	Unrestless of the injured
			The urge to rescue the injured
			Severity of injury
		Emotional atmosphere	Inability to manage emotions
			Stress of bystanders
			Emotion of relief

### Scene shock

In some rare scenes of traffic accidents, there are situations where the injured and bystanders fall into a state of instability and unawareness, identified as the “scene shock.” The existing emotional condition, desperation, and rescue efforts were classified in the “scene shock” category. A breakdown of these quoted concepts is explained next

• Emotional atmosphere: It has been defined as a state of eagerness to help the victim at the accident scene following crowd development, and the efforts of people present at the scene to rescue the victims. People present at the scene, and the companions of the victims, observing the unstable condition of the injured, begin to shout, scream, and cry. One of the contributors who accompanied the injured at the accident scene mentioned the following:

H4 (a 26-year-old male): “At that moment, my mother told me she felt her hands and feet cold and with no sense. I was very anxious. I felt terrible and recalled the accident scene of my uncle, who passed away. I thought my mother was about to die.”

The unstable condition of the injured person was among the main reasons for the severity of the condition foreclosing the ability to make wise decisions from bystanders.

• Desperation or hopelessness is another concept classified in the “scene shock” category. Being in a situation that has not been experienced has put the injured in a halt. The disquiet and restlessness of the injured and the request for help from him/her increase such desperation. The unawareness about first aids imposes an increasing level of anxiety to bystanders. A companion who was the spouse of an injured person, described the accident moment and scene as follows:

H1 (a 27-year-old male): “I do not know how to describe those feelings to you, it feels like you have a dream at that moment, but that is not true. My spouse said that she could not feel her legs and thought they are going to be amputated, which aggravated my anxiety and agitation. I called the emergency and waited for help. At some point, I thought my spouse was dead.”

• The rescue effort was also another concept relating to scene shock. This suggests that the present stress and excitement of the scene result in the life-saving of the injured by companions. This occurs when the situation is unstable, and, at any moment, there is the possibility of another event, like a car explosion. In the same vein, a contributor, as a mother of an injured person, declared the following:

H2 (a 50-year-old female): “I felt so bad, I thought my kids were crumbling, and I was afraid that the car would catch fire. I kept them alive by moving them outside the car. I had seen in the movies that the car might catch fire after the accident. That is why I was so afraid.”

Sometimes, bystanders also blame themselves for the accident, which increases the anxiety and resilience to save the injured. Therefore, they try to save the injured and take the injured to a health center as soon as possible. One of the contributors mentioned the following:

H3 (a 28-year-old female): “I just remember when I opened my eyes, everything, including the car, was crushed. At that moment, the only thing I thought of was to take the kids outside the car. I was the driver, and I caused the incident.”

### Emotional intervention

The next category extracted from the current study was emotional intervention. On this basis, interventions and decisions are made by companions and bystanders at the accident scenes. As a source of emotion, not only they are not beneficial to the injured but also lead to complications, including secondary damages. Moreover, the four extracted concepts were unplanned interventions, emotional behaviors, emotional decision-making, and emotional involvement that generally explained the emotional intervention. Each concept alongside quotations supporting the findings is described below.

• Unplanned interventions: The companions and bystanders’ measures take place in an emotional atmosphere. Such interventions occur following a crowded scene development and the efforts of people present at the scene to rescue the injured people. The prostration was caused by the lack of knowledge on the fate of the injured person and the anticipatory outcomes, the fear of death. Moreover, the insistence on rescue has caused the people to act in an unplanned stage. To this end, one of the contributors confirming the above-mentioned points discoursed the following:

C9 (a 20-year-old male): “As people have no knowledge, they only help with sympathy and fear that the car would catch fire and other terrible events. They just pull the victims outside the car; they do not know how to do it and fail to examine bones in terms of being broken. They just think about taking you away”.

In many cases, the injured person is transferred to the hospital before the medical emergency services arrive. The basic principles of carrying the injured are not observed in this kind of transfer, which includes the fixation of the vertebral column in all the suspected cases of SCI.

• Emotional behavior is also considered as the reaction of scene bystanders indicating violence against the first responders, insistence on the fast rescue, and transferring the injured person, even though pulling the victims from the vehicle exacerbates the damage to the spinal cord. A patient with SCI has mentioned the following:

C5 (a 36-year-old female): “Many passengers were healthy and pulled themselves outside the vehicle through doors or windows. Because of the pressure on my stomach and chest, I suddenly lost my breath, and people were saying that I was bruised. The healthy passengers pulled me out by any possible means. I remember a gentleman asking me to move my foot, but I said I could not”.

• Emotional decisions: Making immediate and illogical decisions is a result of emotional behavior; this is majorly influenced by our momentary feelings. Such emotional decisions are resulted from observing the undesirable condition of the injured, bloodshed, and fractures. Bystanders may lack knowledge about the correct principles of triage and injury assessment; thus, they may evaluate the situation as critical and make illogical decisions.

H3 (a 28-year-old female): “My child was bleeding. I did not dare to approach my child. He was crying and begging me to take him out. I assumed that if I let him remain there for a minute, he would die. A man told me not to worry and that they are going to take him out. He told me “do not wait for the emergency, as they are never on time.”

Some of the decisions mentioned above included not contacting the pre-hospital emergency and transferring the injured with private cars. Moreover, since the basic principles of safe transfer were not observed, the injured experienced secondary damages. One of the injured, who was taken to the hospital by a personal vehicle as a result of decisions of bystanders stated the following:

C9 (a 20-year-old male): “After taking me out of the car, they decided to take me to the hospital with a personal car. They could not move me properly because I am tall. I remember one laid me on the car’s back seat, and one of them went to the other side to pull me. I felt dizzy and was in pain. I remember a guy sitting on the back seat and putting my head on his legs.”

• Emotional involvement was another concept defining emotional intervention and related to the involvement of bystanders and laypeople. It indicates that the laypeople at the scene began to intervene as a result of a sense of humanitarianism, which only resulted in confusion at the scene and prevented the care and triage of the first responders. One of the victims of the incident remarked about the intervention of laypeople in his experience, as follows:

C10 (a 35-year-old male): “Because the car was rolled over and the switch was open, it continued igniting and burning. The people were scared that the car might catch fire. Therefore, one took my hand and pulled me out of the car on the ground getting a few meters away from the car. At that moment, I felt severe pain in my body. Numerous people tried to help me, and some also harmed me. We, Iranians, try to help the injured following accidents, but we do not know how to do so.”

The crowd in the scene of traffic accidents is among the matters that distinguish pre-hospital services from other types. The people who gather at the scene to assist or sometimes only observe could negatively affect the timing and quality of providing timely and appropriate pre-hospital services.

## Discussion

The present study investigated the qualitative circumstances of SCIs in traffic accidents. The obtained results suggested emotional interaction as the leading cause of injury in pre-hospital care. The two concepts of scene shock and emotional intervention explained the more abstract concept of emotional interaction. The scene of traffic accidents is a unique site. The stress and anxiety associated with the scene and the shock created by bystanders, especially companions, prevent rational decision-making. The individuals’ interactions take place in an emotional atmosphere, highlighting the importance of making emotional decisions.

Moreover, the involvement of laypeople and bystanders and their willingness to help, disrupt the work of rescuers and complicate the situation. As a result, secondary harms are imposed on the injured. The initial management of an injured person with a potential SCI initiates at the incident scene. Studies indicated that pre-hospital management for injured patients with SCIs reduces the incidence of neurological defects and prevents any secondary damages to their neurological system [8].

One of the extracted concepts was the “scene shock,” described by three concepts: emotional atmosphere, desperation, and rescue efforts. With regards to the emotional atmosphere at the scene of traffic accidents, excessive fear and anxiety, desperation, helplessness, and uncontrolled emotions are the first reactions of present individuals, called the “scene shock.” Efforts to save the injured person’s life and the fear of death are the main reasons for the anxiety and frustration in companions. When the injured person is locked up in the car, and the emergency staff is delayed, the death anxiety overcomes and urges the companions to pull the injured out of the car, which adds to the desperation. Similar studies indicated that in a traumatic event where the injured suffered from unstable conditions, the companions’ reactions are influenced by fear, stress, and panic [19-21]. Hathaway et al. stated that aggression, guilt, and stress are common emotional responses by individuals during traumatic events [19].

Another category extracted in this study was the emotional intervention classified in four sub-themes: illogical intervention, emotional behaviors, emotional decisions, and emotional involvement. At the incident scene, the bystanders, as well as the injured person, act hastily and perform unnecessary interventions due to the lack of sufficient knowledge of caring. Moreover, stress caused by such unawareness leads to the non-secure transfer of the injured. The lack of protection, improper movement, hasty transfer, and the lack of fixing the body of injured are examples of illogical actions and inappropriate interventions by the bystanders. Khorasani Zavareh reported that laypeople gather at the scene of an incident earlier than the emergency staff and often have anxiety and stress, causing problems and difficulties for the emergency personnel. Moreover, they occasionally impose secondary damages or even mortality to the injured and companions [13].

Notably, they usually take the injured outside the scene and transfer them to the hospital with their cars. Furthermore, according to Ali Nia and Khorasani, improper activities and inappropriate interventions cause even more injuries to the victim [12, 22]. Additionally, Toscano et al. argued that 26% of injured patients suffering from SCIs have experienced worsened neurological status during the transfer to the hospital. The authors have also reported the early causes that exacerbate neurological injuries by the improper movement and non-immobilization of the spinal cord after a traumatic injury [23]. The emotional reaction of laypeople, which is a reflection of the emotional atmosphere of the scene and a hasty attempt to save the lives of the patients, have caused secondary and unintended damages to the injured.

The urge to rescue the injured person was also derived from the present study as one of the concepts of emotional intervention. When the injured person is locked up in the car, and the emergency medical personnel are delayed, the fear of death overcomes and urges those at the scene to pull him/her outside the car. Meanwhile, in case there are other injured people in the car, and they can pull themselves out, the urge to rescue will increase. Similarly, Franzen et al. studied patients that were affected by traffic accidents and showed that the excitement and anxiety of the injured at the accident scene were reduced by the presence of professional caretakers. That included medical emergency experts, which in turn improved trust and safety., the patients feeling more secure until transferred by an ambulance [24]. Anxiety, restlessness, the stress created by the scene, and the emotional atmosphere lead to desperation; this was the next concept derived from the study results. Poursheikhian et al. also stated that severe damage to the injured person causes anxiety and stress in the relatives and companions, leading to the occurrence of uncontrolled and unpredictable behavior like violence and aggression [25]. According to Heidari et al., anxiety and stress have other consequences as well. Sometimes the confusion and anxiety of laypeople in the scene of incident lead to calling numerous ambulances, their number turning out to be higher than needed. Consequently, the scene and may become more crowded and disrupt the arrival of the first aid experts at the incident scene [26]; their finding was in line with the present study.

As defined by the WHO, the tasks of laypeople present at the scene are contacting the pre-hospital emergency and asking for help; securing the scene (to prevent more accidents, prevent the bystanders from being injured and control the crowd of the scene); organizing people and resources; keeping bystanders away from injured people so that first responders can take the necessary actions; help extinguishing the fire; providing first aid, and transferring the injured to the hospital if the ambulance is unavailable [27]. However, one of the most critical issues in the traffic accident scenes is the intervention of laypeople and the lack of knowledge and skill in managing the accident’s situation and victims [13]. In the present study, the involvement of laypeople in the incident scene has led to disruptions and difficulties for the rescuers. Similarly, Ali Nia et al. and Haghparast et al. found that setbacks like people’s involvement in the accident scene, increased the workload of the medical staff, and actions related to the injured and management issues. These issues included insufficient communication technology in accessing proper pre-hospital services by injured patients in the post-accident phase; this could increase the mortality and disability rate of the victims [11, 12]. According to studies conducted in Iran, the emotional reactions from the laypeople present at the scene were associated with anger and the verbal conflicts of people with the first responders. These were some outcomes of the presence of people at the incident scene [25, 28, 29].

In the present study, some injured subjects with SCIs were not interviewed due to stress. This is because of the occurrence of flashbacks and being drawn back into the traumatic experience. Furthermore, most of the interviews were conducted in the hospital and rehabilitation centers; therefore, the patients had to undertake occupational therapy and physiotherapy. This was one of the issues that could interfere with the interview process. In such situations, the interviewer had to continue the interview after performing the rehabilitation activities; however, some patients were unable to continue the interview or focus due to fatigue.

## Conclusion

The obtained results suggested that emotional interaction is a concept that has been repeatedly observed in the scene of traffic accidents. It results in emotional behavior, disrupting the rescuers, hasty actions by bystanders, the improper movement of the injured, and interventions that, in turn, will result in secondary damages to the patient. The emotional atmosphere of the scene and stress level of the injured and the companions, as well as the insistence of the injured person to escape from the harsh condition, have caused those lacking medical knowledge and expertise to unsafely transfer the patient and cause secondary damages, like aggravating SCIs or even causing them. Providing training to the emergency staff as well as laypeople through media can reduce the negative emotions to some extent, and improve the patient’s management. Further studies are required to conduct more precise evaluations in order to design a protocol on the management of accident scenes. Researching by a grounded theory approach is recommended for a more in-depth investigation of the scene of the incident and the development of SCIs in the injured, as well as the design of a relevant protocol.

## Conflict of Interest

The authors confirm that there are no conflicts of interest.
